# Resolution Agonist 15-epi-Lipoxin A_4_ Programs Early Activation of Resolving Phase in Post-Myocardial Infarction Healing

**DOI:** 10.1038/s41598-017-10441-8

**Published:** 2017-08-30

**Authors:** Vasundhara Kain, Fei Liu, Veronika Kozlovskaya, Kevin. A. Ingle, Subhashini Bolisetty, Anupam Agarwal, Santosh Khedkar, Sumanth D. Prabhu, Eugenia Kharlampieva, Ganesh V. Halade

**Affiliations:** 1 Division of Cardiovascular Disease, The University of Alabama at Birmingham, Alabama, USA; 2 Department of Chemistry, The University of Alabama at Birmingham, Alabama, USA; 3 Division of Nephrology, The University of Alabama at Birmingham, Alabama, USA; 4 ChemBio Discovery, Inc., Lexington, Massachusetts, USA

## Abstract

Following myocardial infarction (MI), overactive inflammation remodels the left ventricle (LV) leading to heart failure coinciding with reduced levels of 15-epi-Lipoxin A_4_ (15-epi LXA_4_). However, the role of 15-epi LXA_4_ in post-MI acute inflammatory response and resolving phase is unclear. We hypothesize that liposomal fusion of 15-epi-LXA_4_ (Lipo-15-epi-LXA_4_) or free 15-epi-LXA_4_ will expedite the resolving phase in post-MI inflammation. 8 to 12-week-old male C57BL/6 mice were subjected to permanent coronary artery ligation. Lipo-15-epi-LXA_4_ or 15-epi-LXA_4_ (1 µg/kg/day) was injected 3 hours post-MI for (d)1 or continued daily till d5. 15-epi-LXA_4_ activated formyl peptide receptor *(FPR2)* and *GPR120* on alternative macrophages but inhibited *GPR40* on classical macrophages *in-vitro*. The 15-epi-LXA_4_ injected mice displayed reduced LV and lung mass to body weight ratios and improved ejection fraction at d5 post-MI. In the acute phase of inflammation-(d1), 15-epi-LXA_4_ primes neutrophil infiltration with a robust increase of Ccl2 and FPR2 expression. During the resolving phase-(d5), 15-epi-LXA_4_ initiated rapid neutrophils clearance with persistent activation of FPR2 in LV. Compared to MI-control, 15-epi-LXA_4_ injected mice showed reduced renal inflammation along with decreased levels of *ngal* and plasma creatinine. In summary, 15-epi-LXA_4_ initiates the resolving phase early to discontinue inflammation post-MI, thereby reducing LV dysfunction.

## Introduction

Myocardial infarction (MI) is the leading cause of heart failure and is responsible for a high number of mortalities resulting from persistent and unresolved inflammation^1^. In heart failure pathology, there has been displayed a consistent increase in the number of cytokines and chemokines that are the hallmark of chronic inflammation and kidney dysfunction^2^. Traditional inflammation treatment using non-steroidal anti-inflammatory agents (e.g. rofecoxib and celecoxib) was unsuccessful to restore the function of the inflamed infarct area in clinical settings; instead, inhibition of inflammation treatments provoked MI events and kidney pathology^3–5^. Thus, inhibition of inflammation or suppression of cytokines has been an ineffective approach in delaying heart failure post-MI^6^, indicating the diversified role of MI-induced cytokines in heart failure pathology has been overemphasized for decades. In response to myocardial injury, the physiological innate response consists of programing the clearance and healing of the left ventricle (LV)^7^. However, the non-resolving innate response programs the LV towards heart failure^8^. Thus, by exploring an alternative strategy to resolve uncontrolled inflammation presents an important unmet medical need to re-examine methods to delay or reduce heart failure. Post-MI resolution of inflammation is an active phase that limits subsequent LV remodeling and heart failure. Following MI, an overactive neutrophil and macrophage response adversely remodels the LV to alters size, shape, and function with progressive heart failure pathology^9^. Post-MI healing occurs in two phases: the early acute inflammatory phase and the resolving phase (also called late or reparative phase)^10^. The acute phase is marked by the entry of neutrophils and monocytes/macrophages, followed by the rapid repression of pro-inflammatory genes, indicating the resolution of inflammation^1^. Post-MI, the splenic supply of leukocytes correlates with pro-inflammatory circulating leukocytes that are associated with the acute inflammatory response of the LV, marked by the proliferation of monocyte progenitor cells and activation of monocytes^11, 12^. A persistent supply of overactive immune cells post-MI, such as polymorphonuclear leukocytes (PMNs), contributes to the pathology of congestive heart failure^13^. Thus, to resolve the inflamed infarcted area post-MI, the early activation of the resolving phase is essential^13–15^.

Lipoxygenase interaction products (lipoxins; LXs) are endogenous, pro-resolving, lipid mediators generated from membrane arachidonic acid through biochemical synthesis involving the enzymes 5- and 15-lipoxygenase (5-LOX and 15-LOX). The primary function of LXs is to coordinate the functions and recruitment of PMNs to promote clearance of debris^16^. The major pathway for generating LXs is augmented in the presence of aspirin, through cyclooxygenase (COX)-2 and 5-LOX activity during the acute inflammatory response^17^. In order to offer pharmacological action, 15-epimer LXA_4_ (15-epi-LXA_4_) binds to the G-coupled protein receptor FPR2^18, 19^. Endogenous natural lipoxin A_4_ also inhibits TGF-β1–dependent collagen secretion and α-SMA expression in human lung myofibroblasts^20^. Importantly, 15-epi-LXA_4_ levels were lower than normal values in patients experiencing the progressive development of chronic heart failure due to the defective resolution of inflammation^21^. Here, we hypothesize that the stable form of LXA_4_; 15-epi-LXA_4_ with liposomal fusion (Lipo-15-epi-LXA_4_) or free 15-epi-LXA_4_ would initiate the resolving phase at the precise time in post-MI healing to limit cardiac remodeling and subsequent heart failure. To address the instability of the hydrophobic compound, 15-epi-LXA_4_ was incorporated into a liposomal fusion^15^. Our *in-vitro* and *in-vivo* results indicate that post-MI treatment of pure 15-epi-LXA_4_ or liposomal 15-epi-LXA_4_ initiates the early resolving phase, suggesting a therapeutic potential of the lipoxin biomolecule to delay MI-induced cardiorenal pathology.

## Results

### 15-epi-LXA_4_ activated *FPR2*, and *GPR120* but inhibits *GPR40* on pro-resolving macrophages

**Figure 1:**
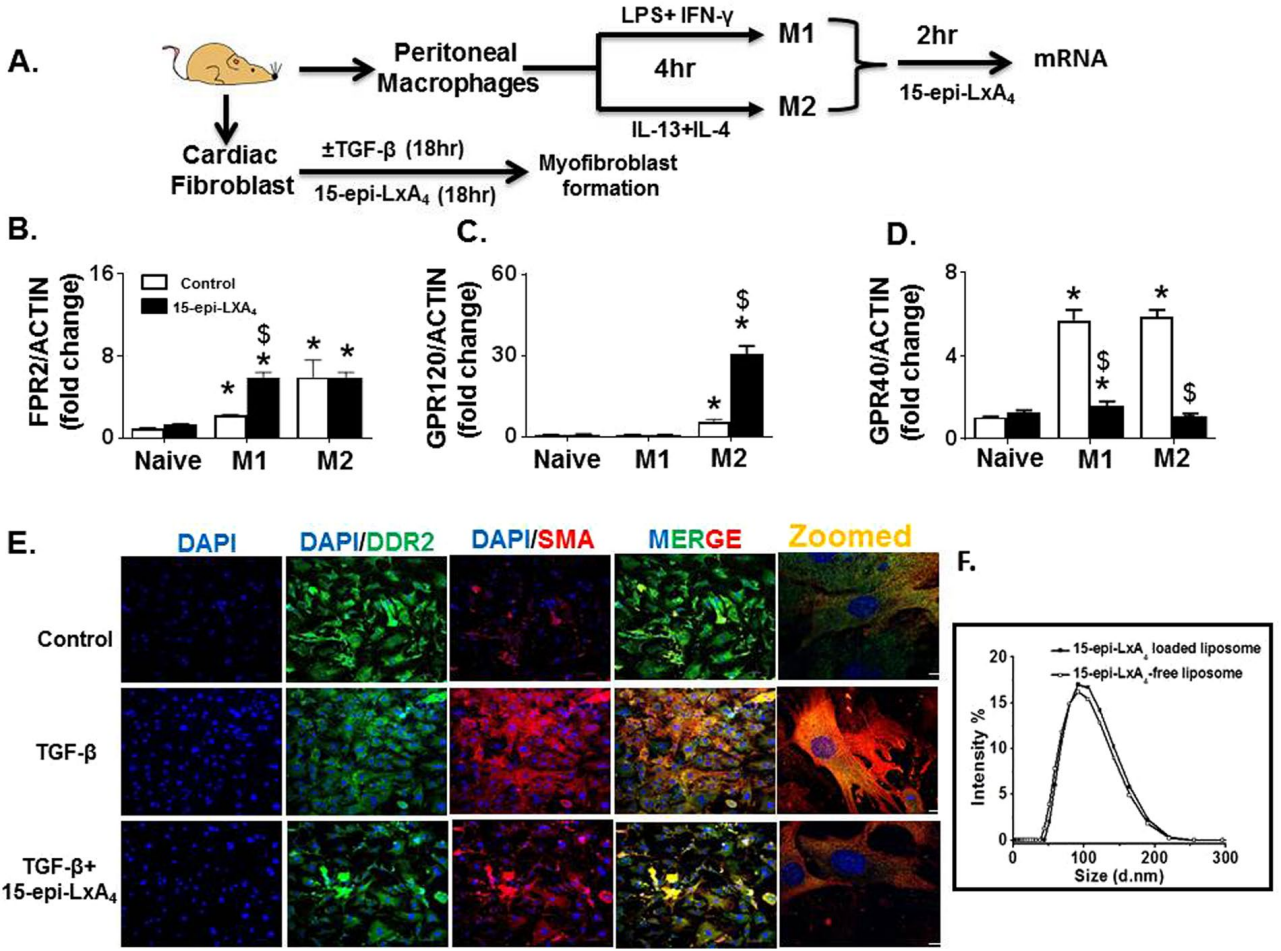
15-epi-LXA_4_ upregulates macrophage FPR2 and GPR120 and limits TGF-β-mediated myofibroblast differentiation. (**A**) *In-vitro* study design using peritoneal macrophages stimulated with LPS (1 µg/ml) + IFNγ (20 ng/ml) to differentiate in M1 macrophage or IL-4 (20 ng/ml) + IL-13(20 ng/ml) to differentiate into M2 macrophage and cardiac fibroblast isolated from male C57BL/6 mice (6–8 weeks of age) treated with TGF-β for myofibroblast differentiation. mRNA expression of (**B**) *FPR2* (**C**) *GPR12*0 (**D**) *GPR40* on proinflammatory (M1) and proresolving (M2) peritoneal macrophages with and without 15-epi-LXA_4_ (100 nM) treatment. *p < 0.001 vs. naïve macrophages, ^$^p < 0.05 vs 15-epi-LXA_4_ treatment. Data represents n = 5 independent experiments done in a replicate of three/treatment. (**E**) Immunofluorescence representative images showing DDR2 and α-SMA expression in myofibroblast differentiated using TGF-$$\beta $$ (15 ng/ml) with and without 15-epi-LXA_4._ Data is representative of n = 3 experiment done independently. (**F**) Intensity curve displaying stable size of liposomal 15- epi-LXA_4_.

To determine which mature phenotypes of macrophage expresses FPR2, GPR120, GPR40 receptors, and to further understand the response of 15-epi-LXA_4_ on these receptors, the peritoneal macrophages were used. A cocktail of LPS and IFN-γ differentiated peritoneal macrophage (PM) into the pro-inflammatory (M1) phenotype; while a mixture of IL-13 and IL-4 differentiated the alternative or pro-resolving (M2) phenotype and then examined the effect of 15-epi-LXA_4_ on differentiated macrophages. In response to M1 and M2 simulative cocktails, both M1 and M2 macrophage displayed similar expression of *(formyl peptide receptor 2) FPR2*, a GPCR receptor protein known to bind with LXA_4﻿(Fig. 1A)._ Co-incubation and stimulation with 15-epi-LXA_4_ (100 nM) upregulated *FPR2* expression on M1 type macrophage (Fig. 1B). Inversely, *GPR120*, (GPCR receptor) expression was stimulated on M2 macrophage and 15-epi-LXA_4_ (100 nM) amplified the expression of *GPR120* only on M2 macrophage (Fig. 1C). In contrast, *GPR40* (GPCR receptor) was highly expressed on both M1 and M2 macrophages; however, treatment with 15-epi-LXA_4_ (100 nM) for 2 hrs inhibited the expression of *GPR40* on both M1 and M2 macrophages (Fig. 1D). Thus, our *in-vitro* results indicated that 15-epi-LXA_4_ serves not only as a ligand for a *FPR2* receptor, but also regulates fatty acid sensors *GPR40* and *GPR120* that polarize the macrophage phenotype.

### 15-epi-LXA_4_ limited TGF-β induced myofibroblast formation in cardiac fibroblast *in-vitro*

The transition of fibroblast to secretory myofibroblast leads to collagen deposition and is involved in the pathophysiological process of scar formation in post-MI healing. To test 15-epi-LXA_4_, we differentiated cardiac fibroblast into myofibroblast using TGF-β. As shown in Fig. 1E, TGF-β induced fibroblast hypertrophy within 18 hours transitioning fibroblast into myofibroblast. The formation of myofibroblast is indicated by higher expression of α-SMA along with DDR2 and having a typical myofibroblast phenotype. Co-incubation of 15-epi-LXA_4_ (100 nM) with TGF-β (15 ng/ml) for 18 hours reduced α-SMA expression. Cultured fibroblast treated with 15-epi-LXA_4_ were less stellate and became more spindle shape, displaying a significant decrease in stellate:spindle cell ratio compared with TGF-β treated cells, thereby having relatively similar morphology to fibroblasts (Fig. 1E and Supplementary Figure 1). Thus, 15-epi-LXA_4_ limited TGF-β induced hypertrophy as well as the differentiation of fibroblast into myofibroblast *in-vitro*.

### Quality control outcome for 15-epi-LXA_4_ liposomes

EPC/DSPE-PEG (2000) liposomes were investigated here to achieve a stable 15-epi-LXA_4_ loaded system by overcoming the natural unstable properties of the liposome. The attached PEG chain can stabilize the drug loaded liposomes for storage up to 300 days at 2 °C to 8 °C^22^. Instead of using passive and active loading methods, 15-epi-LXA_4_ was loaded in the liposomes by using thin layer dehydration method to achieve higher encapsulation efficiency. As illustrated (Fig. 1F), the average particle size of blank liposome was 87.80 nm with a polydispersity index (PDI) of 0.096 after extrusion, indicating the monodisperse distributions based on this formulation. For extruded 15-epi-LXA_4_ liposomes, the average size slightly increased exhibiting at 93.75 nm with the PDI at 0.086, which also confirms the monodispersity for 15-epi-LXA_4_ loaded liposomes. The low PDI of the liposomes resulted from the uniform incorporation of 15-epi-LXA_4_ and the extrusions (Fig. 1F)^23, 24^.

### 15-epi-LXA_4_ treated mice attenuated LV dysfunction and reduced pulmonary edema post-MI

**Figure 2:**
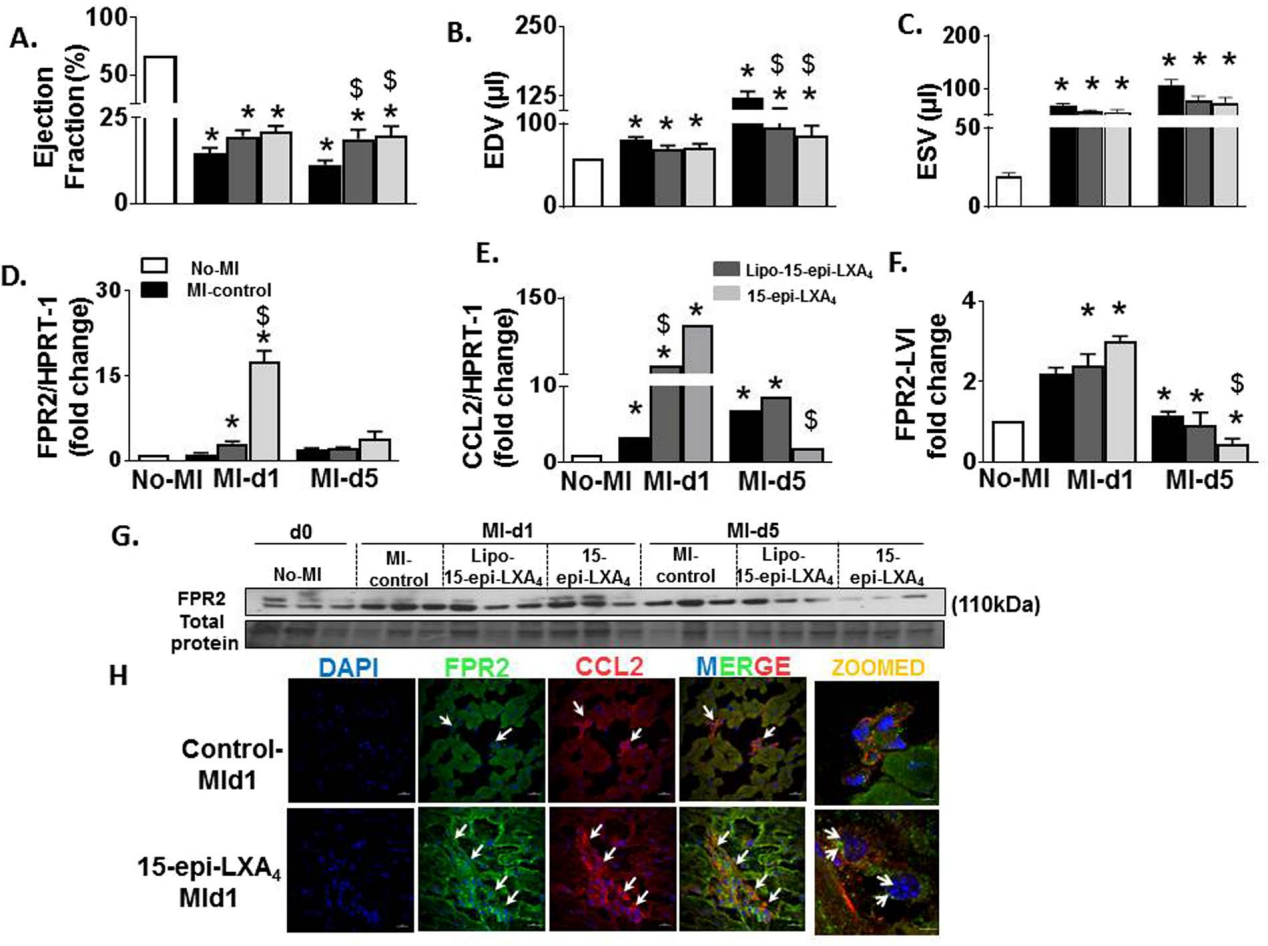
15-epi-LXA_4_ mediates resolution of inflammation via activation of FPR2 and Ccl2 and improving LV function. Graphs representing echocardiographic data (**A**) Ejection fraction (**B**) End diastolic volume (EDV) (**C)** End systolic volume (ESV). n = 5–9 mice/group/day *p < 0.01 vs. d0, ^$^p < 0.05 vs MI-control at respective day. (**D**,**E**) mRNA expression of *FPR2* and *Ccl2* in LVI of No-MI, MI-control, Lipo-15-epi-LXA_4_ (1 µg/kg) and 15-epi-LXA_4_ (1 µg/kg) injected mice at post-MI d1 and d5. n = 5 mice/group/day *p < 0.001 vs. d0, ^$^p < 0.05 vs MI-control at respective day. (**F**) Densitometric analysis of FPR2 immunoblot. n = 3 mice/group/day *p < 0.001 vs. d0, ^$^p < 0.05 vs MI-control at respective day. (**G**) Immunoblot is representing FPR2 expression in LVI. (**H**) Representative immunofluorescence LVI images showing FPR2 (green) and Ccl2 (red) expression in MI-control and 15-epi-LXA_4_ injected mice, (magnification 40x). Data is representative of n = 3 experiment done independently.

Echocardiography measurements were performed to assess the effect of 15-epi-LXA_4_ treatment on LV function post-MI. Echocardiography data (Fig. 2A) showed higher, ejection fraction (19% (Lipo 15-epi-LXA_4_) and 18% (15-epi-LXA_4_); p < 0.05) with improved EDV (end-diastolic volume and ESV (end systolic volume) in 15-epi-LXA_4_ treated mice compared with MI control mice at d5 post-MI (Fig. 2B and C). The 15-epi-LXA_4_ treated mice showed a trend of improvement in fractional shortening, EDD (end-diastolic dimension), ESD (end-systolic dimension) and IVSd (interventricular septal end diastole) compared with MI-control mice. Both Lipo- and free 15-epi-LXA_4_ treated mice showed reduced hypertrophy with a decrease in LV/Body weight ratio (4.0 ± 0.10 vs. 4.8 ± 0.12) compared to MI-control mice (Table 1).

**Table 1:** Echocardiography measurements in 15-epi-LXA_4_ treated and MI-control mice post-MI.

Parameters/groups	d0 naïve control	MI-day 1			MI-day 5		
		MI-control	Lipo-15-epi-LXA_4_	15-epi-LXA_4_	MI-control	Lipo-15-epi-LXA_4_	15-epi-LXA_4_
n	6	9	8	7	9	6	5
Heart rate (bpm)	433 ± 14	450 ± 9	464 ± 9	480 ± 13	440 ± 12	504 ± 20	485 ± 13
EDD (mm)	3.6 ± 0.1	4.8 ± 0.2*	4.4 ± 0.1*	4.3 ± 0.2 *	5.6 ± 0.1*	5.4 ± 0.4*	5.0 ± 0.3*^#^
ESD (mm)	2.4 ± 0.1	4.4 ± 0.1*	4.0 ± 0.1*^#^	3.9 ± 0 0.2*^#^	5.3 ± 0.1*	4.9 ± 0.5*^#^	4.6 ± 0.4*^#^
Fractional shortening %	35 ± 2	8 ± 1*	10 ± 1*	10 ± 1*	6 ± 1*	10 ± 2*^#^	8 ± 2*
PWTs (mm)	1.04 ± 0.05	0.54 ± 0.03*	0.59 ± 0.04*	0.57 ± 0.04*	0.54 ± 0.04*	0.58 ± 0.07*	0.52 ± 0.03*
IVSs (mm)	1.0 ± 0.02	0.65 ± 0.05*	0.63 ± 0.04*	0.62 ± 0.05^*^	0.55 ± 0.03*	0.74 ± 00.01*^#^	0.57 ± 0.09*
PWTd(mm)	0.65 ± 0.04	0.48 ± 0.04*	0.52 ± 0.04*	0.50 ± 0.04*	0.45 ± 0.03*	0.48 ± 0.05*	0.50 ± 0.02*
IVSd (mm)	0.71 ± 0.03	0.55 ± 0.03*	0.55 ± 0.03*	0.52 ± 0.03^*^	0.50 ± 0.03*	0.61 ± 0.02*^#^	0.50 ± 0.02*

Values are mean ± SEM; n indicates sample size. bpm, beats per minute; EDD, end-diastolic dimension; ESD, end-systolic dimension; PWT, Posterior wall thickness; IVSs, Interventricular septal end systole; IVSd, interventricular septal end diastole; mm, millimeter; µl, microliter. *p < 0.05 vs. day 0 naïve control; ^#^ *p* < 0.05 vs. MI-control with respective time point.

Gravimetric analyses indicated reduced lung pulmonary edema in 15-epi-LXA_4_ treated mice with a decrease in lung mass-to-body weight ratios compared to MI-control at d5 post-MI (Table 2). Thus, reduced pulmonary edema decreased LV dilation and increased ejection fraction leading to improved LV function in 15-epi-LXA_4_ treated mice post-MI.

**Table 2:** Necropsy parameters in 15-epi-LXA_4_ treated and MI-control mice post-MI.

Necropsy parameters	d0 naïve control	MI-day 1			MI-day 5		
		MI-control	Lipo-15-epi-LXA_4_	15-epi-LXA_4_	MI-control	Lipo- 15-epi-LXA_4_	15-epi-LXA_4_
n	6	9	8	7	10	6	5
Body weight (g)	23 ± 1	26 ± 2	25 ± 1	23 ± 1	20 ± 6	27 ± 1	23 ± 1
LV (mg)	73 ± 2	87 ± 4*	83 ± 4*	83 ± 4*	95 ± 4*	106 ± 5*	91 ± 6*
LV/BW (mg/g)	3.3 ± 0.2	3.4 ± 0.1	3.4 ± 0.1	3.6 ± 0.1	4.8 ± 0.12	4 ± 0.2	4 ± 0.3
Right ventricle (mg)	17 ± 1	18 ± 1	19 ± 1	18 ± 1	20 ± 1	23 ± 2*	20 ± 1
RV mass/ BW	0.8 ± 0.04	0.7 ± 0.1*	0.8 ± 0.04	0.8 ± 0.02	1.1 ± 0.04*	0.9 ± 0.1^#^	0.9 ± 0.1^#^
Lung mass mg/ W body weight (g)	8 ± 0.2	6 ± 0.7	8 ± 0.9	6 ± 0.6	10 ± 0.5*	6 ± 0.3^#^	6 ± 0.3^#^
Tibia (mm)	17 ± 0.1	17 ± 0.1	16 ± 0.1	17 ± 0.1	17 ± 0.1	17 ± 0.2	16 ± 0.3
Infarct area (%)	ND	49 ± 1*	48 ± 1*	49 ± 1*	52 ± 1*	51 ± 2*	50 ± 2*

Values are mean ± SEM; n indicates sample size. LV, left ventricle; BW, body weight *p < 0.05 vs. day 0 naïve control; #p < 0.05 vs. MI-control with respective time point, ND: infarct area not detected in naïve d0 control.

### 15-epi-LXA_4_ activated FPR2 in cardiosplenic and cardiorenal network post-MI

Post-MI 15-epi-LXA_4_ action was determined in the acute and resolving phase, at the site of LV injury and distant organs like spleen and kidney. *FPR2* and Ccl2 expression were measured in the LV, kidney, and spleen. In the acute phase of post-MI healing at d1 both Lipo (2.4 fold) and free 15-epi-LXA_4_ (9.2 fold) injected groups displayed a robust increase of *FPR2*; p < 0.05 in infarcted LV (LVI) compared with MI-control group (Fig. 2D). There was a minimal increase in *FPR2* expression at d1 post-MI in the spleen (Supplementary Figure 2A). The kidney of lipo-15-epi-LXA_4_ injected mice displayed a signifiant increase in *FPR2* (3.8 fold; p < 0.05) expression compared with MI control and 15-epi-LXA_4_ injected mice at d1 post MI (Supplementary Figure 2C). Along with *FPR2*, there was a significant increase in *Ccl2* (MCP-1; monocyte chemotactic protein 1) which recruits monocytes to the sites of injury in LVI, spleen and kidney at d1 post-MI in 15-epi-LXA_4_ injected mice (25-fold; LV, 1200-fold; spleen, 1.2-fold; kidney) compared with MI-control (Fig. 2E and Supplementary Figure 2C and D). During the resolution phase (at d5 post-MI) the LVI and kidney of MI-control, Lipo-15-epi-LXA_4_, and 15-epi-LXA_4_ displayed diminished expression of FPR2 compared with no-MI d0 control. However, the spleen of 15-epi-LXA_4_ showed a 2.4-fold increase in *FPR2* expression compared with MI-control and Lipo-15-epi-LXA_4_ injected mice at d5 post-MI. Increased mRNA expression was confirmed by an increase in protein levels in LVI (Fig. 2F,G). FPR2 activation was confirmed by nuclear translocation in the infarcted LV (Fig. 2H). Of note, in lipo-15-epi-LXA_4_ injected mice, *Ccl2* expression was increased only in the LVI (6.2-fold; p < 0.05) compared to the MI-control group. During post-MI resolving phase at d5, 15-epi-LXA_4_ injected mice displayed the persistent activation of *Ccl2* in the kidney compared with MI-control group. Thus, 15-epi-LXA_4_ primed time-dependent activation of *FPR2-* and *Ccl2*, which is indicative of leukocyte trafficking in the LVI during acute and resolving phase post-MI.

### 15-epi-LXA_4_ promotes neutrophil clearance without affecting acute inflammatory response post-MI

**Figure 3:**
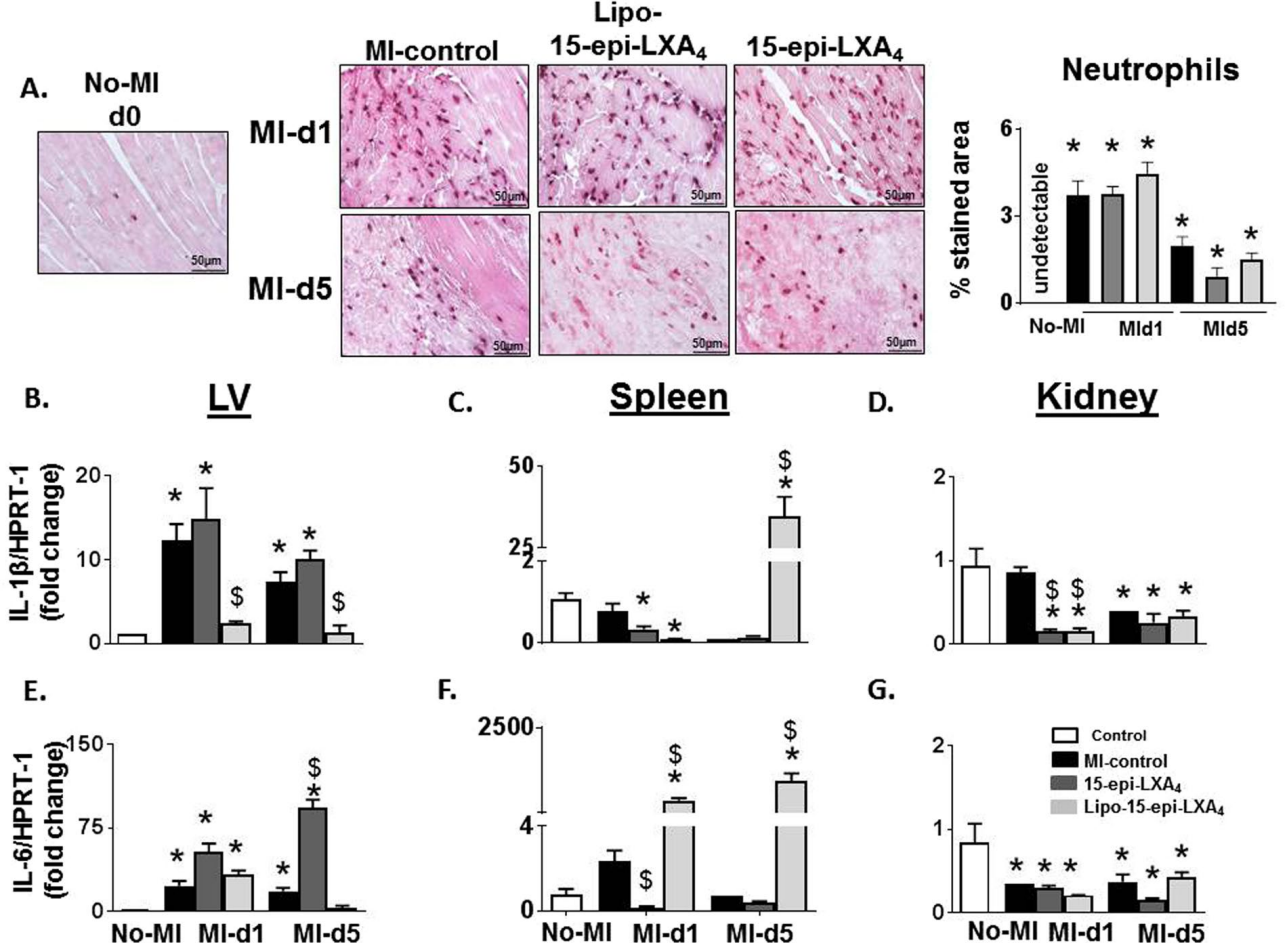
15-epi-LXA_4_ promotes early neutrophil clearance by d5 without altering innate inflammatory phase post-MI. (**A**) Representative neutrophil immunohistochemistry images of LV transverse section of No-MI, MI-control and 15-epi-LXA_4_ (1 µg/kg) injected mice at d1 and d5 post-MI (Magnification 40X, LV middle section at 1.25x, scale = 50 µm). Data is representative of n = 4–5 LV sections/group. mRNA expression of proinflammatory markers (**B–G**) *IL-1β* and *IL-6* in LV, spleen, and kidney in No-MI, MI-control, Lipo-15-epi-LXA_4_ (1 µg/kg) and 15-epi-LXA_4_ (1 µg/kg) injected mice at d1 and d5 post-MI. n = 5 mice/group/day *p < 0.05 vs. d0, ^$^p < 0.05 vs MI-control at respective day.

The recruitment of phagocytic neutrophils to clear myocyte debris is the foremost response of innate immune cells in post-MI injury which peaks during the acute phase (d1) post-MI^25^. Thus, to evaluate the response of the 15-epi-LXA_4_ treatment on neutrophils and on the proinflammatory response, we measured *IL-6* and *IL-1β* along with neutrophil density in the infarcted area. During the acute phase (post-MI d1) neutrophils invaded the infarcted LV in all the groups, both Lipo-15-epi-LXA_4_ and 15-epi-LXA_4_ injected group, exhibited a higher density of neutrophils compared to MI-control samples (Fig. 3A). Of note, neutrophils were rapidly cleared from the infarcted area from both the treated groups during the resolving phase (d5 post-MI) compared to MI-controls. However, the clearance was higher in Lipo-15-epi-LXA_4_ group. Since the neutrophils peaked in Lipo-15-epi-LXA_4_ injected mice, we further measured pro-inflammatory markers *IL-6* and *IL-1β* during the acute and resolving phase. Lipo-15-epi-LXA_4_ and 15-epi-LXA_4_ incited *IL-6* levels with no change in *IL-1β* in LVI in the acute phase (Fig. 3B and E). The spleen and kidneys of 15-epi-LXA_4_ injected mice displayed a decrease in *IL-1β* levels. The spleens of 15-epi-LXA_4_ injected mice showed higher expression of *IL-6* and *IL-1β* compared with MI-saline group (Fig. 3C,D and F,G). Thus, our data showed both Lipo- and 15-epi-LXA_4_ stimulated post-MI neutrophil clearance response in the infarcted LV without altering the acute phase of inflammation.

### 15-epi-LXA_4_ primed resolving phase post-MI

**Figure 4:**
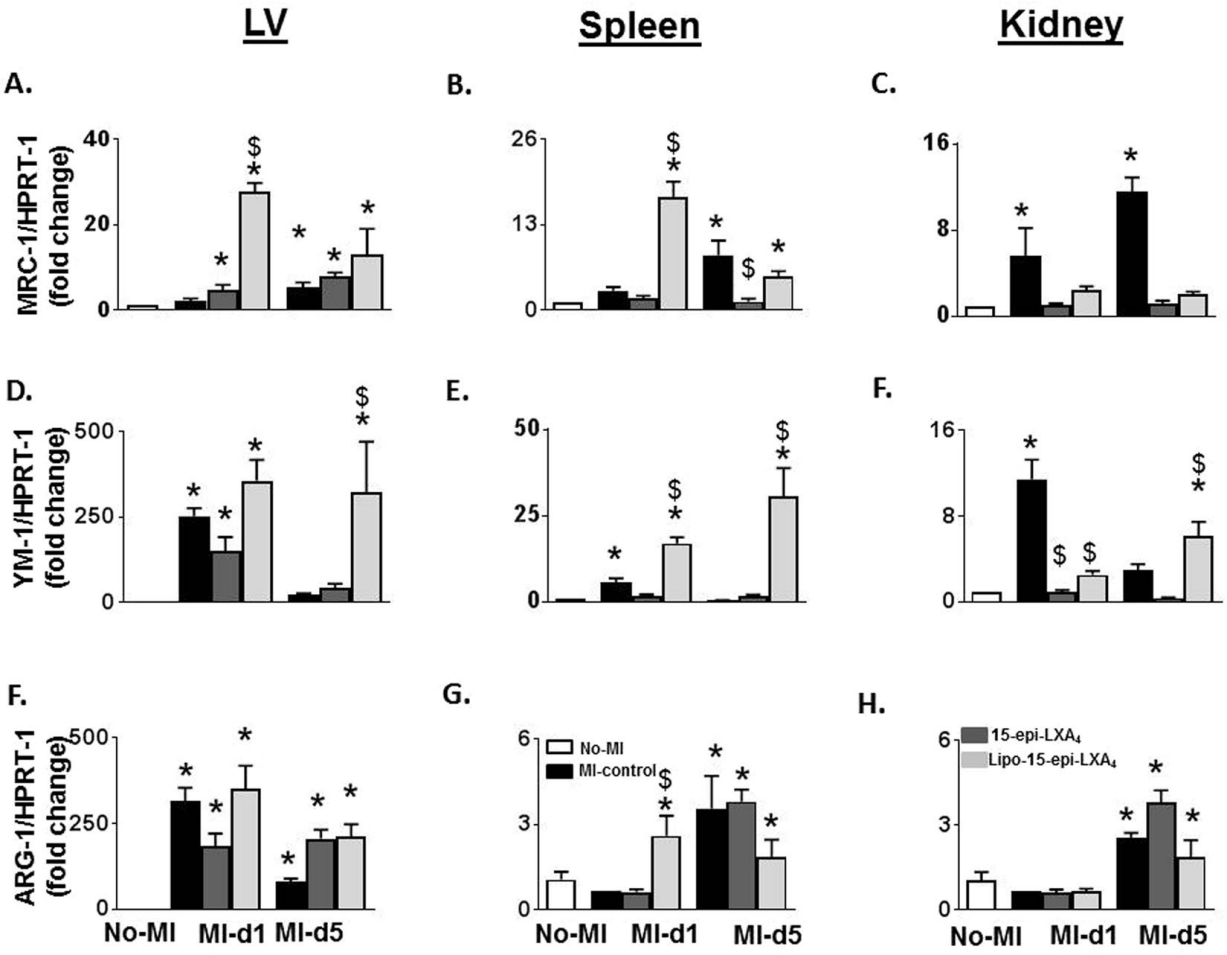
15-epi-LXA_4_ promoted resolving response by activating pro-resolving macrophages in LVI, spleen, and kidney. mRNA expression of (**A**–**H**) *Mrc-1, Ym-1* and *Arg-1* gene expression in LVI, spleen, and kidney in No-MI, MI-control, Lipo-15-epi-LXA_4_ (1 µg/kg) and 15-epi-LXA_4_ (1 µg/kg) injected mice at d1 and d5 post-MI. n = 5 mice/group/day *p < 0.05 vs. d0, ^$^p < 0.05 vs MI-control at respective day.

Since 15-epi-LXA_4_, injected mice displayed an unaltered innate response, next, we determined how 15-epi-LXA4 acts on the post-MI resolving phase. In order to understand its role during the healing phase, we measured gene expression of proresolving markers such as *Mrc-1*, *Ym-1*, and *arg-1* in LVI, spleen, and kidney in the acute and resolving phases. In the acute phase, at d1, *Mrc-1*, *Ym-1*, *and arg-1* expression were increased in LVI of all the three groups compared with no-MI control. *Mrc-1* was elicited in LVI of Lipo-15-epi-LXA_4_ (2.5-fold; p < 0.05) and 15-epi-LXA_4_ (12.3-fold; p < 0.05) group compared to MI-controls (Fig. 4A). In the spleen, 15-epi-LXA_4_ injected mice displayed an increase in transcripts of all three proresolving markers *Mrc-1* (4.6-fold), *Ym-1* (3.4-fold) *and arg-1* (5.1-fold) compared to MI-control and Lipo-15-epi-LXA_4_ injected mice. In the resolution phase (d5), Lipo-15-epi-LXA_4_ and 15-epi-LXA_4_ displayed increases in *arg-1* and *Ym-1* at d5 post-MI (all p < 0.05) in the LV compared with MI-control group. The spleens of 15-epi-LXA_4_ injected mice increased *Mrc-1* and *Ym-1* compared to Lipo-15-epi-LXA_4_ and MI-control, however, no-significant changes were observed in the kidney of 15-epi-LXA_4_ injected group in the acute and resolution phases post-MI (Fig. 4B–H).

### 15-epi-LXA_4_ activated *GPR120* and inhibited *GPR40* in LV during the resolution phase post-MI

**Figure 5:**
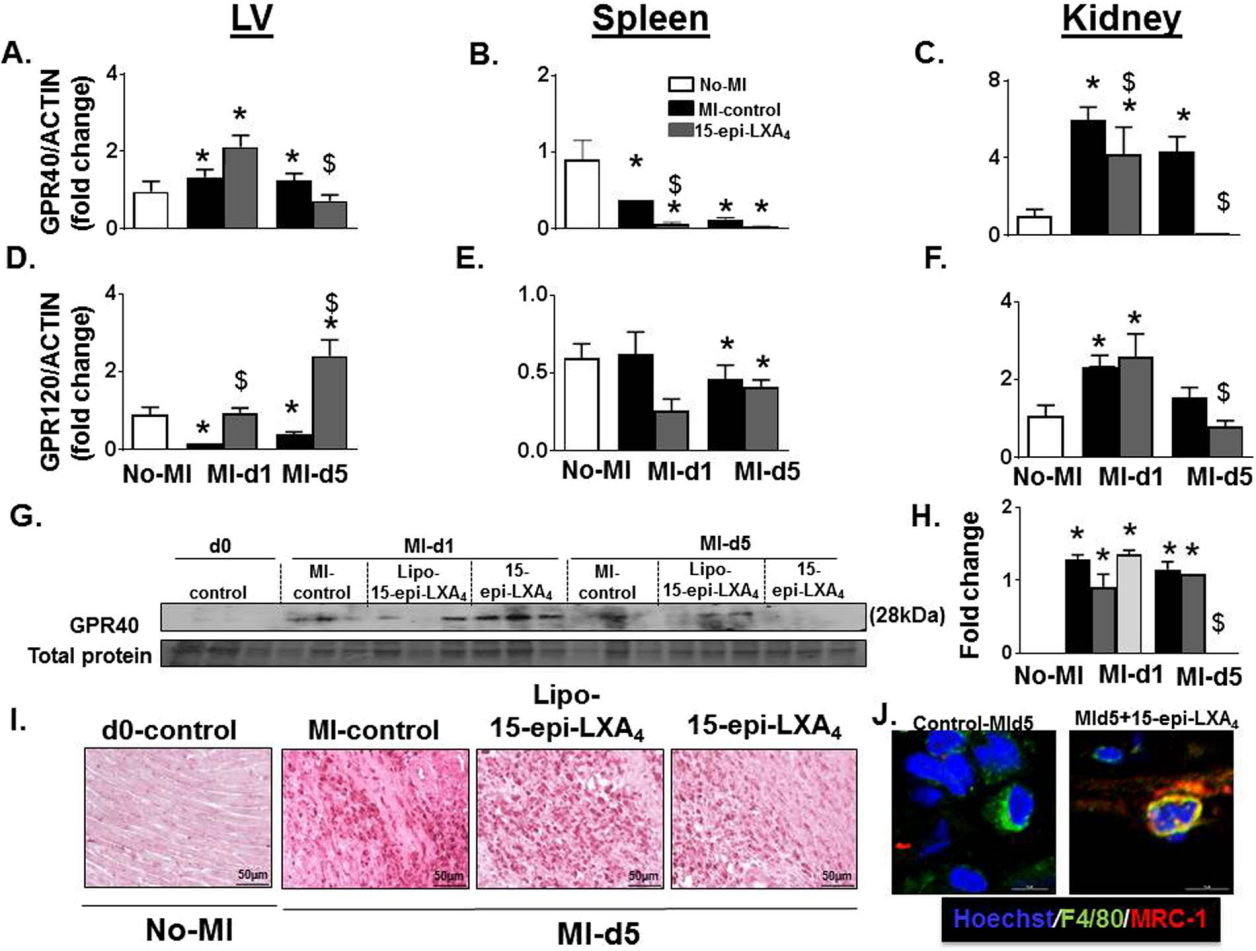
15-epi-LXA_4_ stimulates *GPR120* and decreases *GPR40* in LV post-MI. mRNA expression of **(A**–**F**) *GPR40* and *GPR120* in LVI, spleen, and kidney at d0 control andat d1 and d5 post-MI in MI-control and 15-epi-LXA_4_ (1 µg/kg) injected mice. n = 5 mice/group/day *p < 0.05 vs. d0, ^$^p < 0.05 vs MI-control at respective day (**G**) Immunoblot representing GPR40 expression in LV. (**H**) Densitometric analysis of GPR40 immunoblot. n = 3 mice/group/day *p < 0.05 vs. d0, ^$^p < 0.05 vs MI-control at respective day. (**I**) Representative macrophages immunohistochemistry images of LV transverse section No-MI, MI-control, and 15-epi-LXA_4_ (1 µg/kg) injected mice at d5 post-MI (magnification 40X, LV mid-cavity section at 1.25x, scale = 50 µm). **(J)** Representative immunofluorescence expanded images of LVI showing colocalization of F4/80 (green) and MRC-1 (red) expression in MI-control and 15-epi-LXA_4_-injected mice, (high power field; 60x) at d5 post-MI. Nuclei are stained as blue by hoescht staining n = 2–3 mice/group.

As noticed in the *in-vitro* results, 15-epi-LXA_4_ acts as a ligand for both *GPR120* and *GPR40, we* further investigated whether this validates *in-vivo* post-MI settings. In the acute healing phase (d1) *GPR40* expression increased in the LVI and kidneys but decreased in the spleen post-MI. In the resolving phase (d5) the *GPR40* expression was decreased in all three tissues in MI-control. The 15-epi-LXA_4_ treatment decreased *GPR40* expression in both acute and resolving phase post-MI mice compared with MI-control group (Fig. 5A–C). MI-control mice decreased *GPR120* expression in both the acute and resolving phases in LVI and spleen. In the kidneys, *GPR120* expression was increased in the acute phase (d1) and decreased during the resolving phase (d5) in MI-control group. Higher expression of GPR120 is known for anti-inflammatory effect, here in post-MI setting 15-epi-LXA_4_ activated GPR120 without affecting macrophages density (Fig. 5I and Supplementary 4A). The reparative macrophage phenotype was confirmed by co-localization (yellow) of macrophage surface marker F4/80 (green) and resolving marker MRC-1 (red) (Fig. 5J and Supplementary 3B and 4). Thus, 15-epi-LXA_4_ injected mice displayed an increase in *GPR120* expression in LVI with no change in spleen and decreased expression in the kidney at d5 post-MI (Fig. 5D–F).

### 15-epi-LXA_4_ inhibits acute kidney inflammation

**Figure 6:**
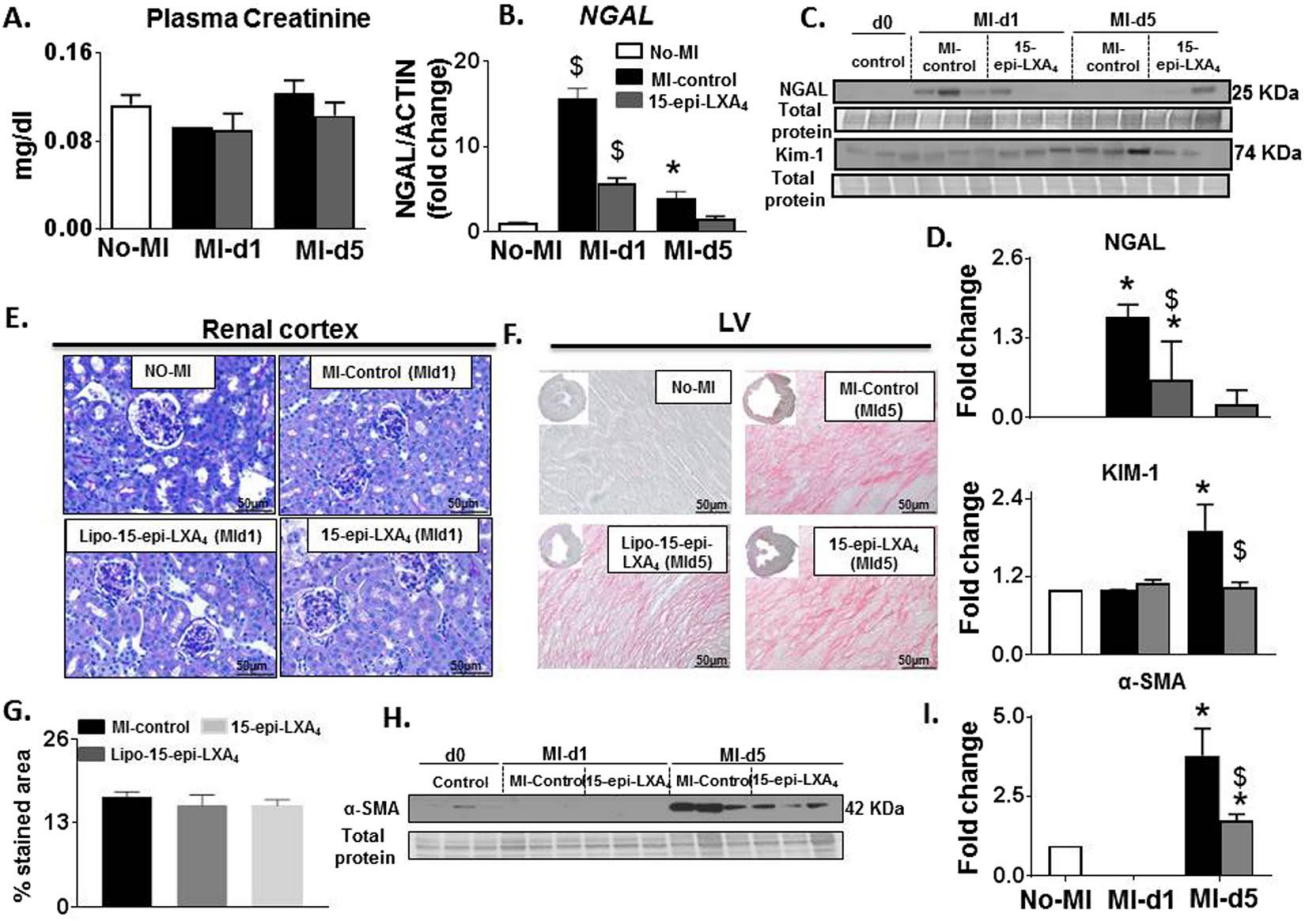
15-epi-LXA_4_ attenuates cardiorenal inflammation at d1 without affecting LV collagen matrix at d5 post-MI. (**A**) Plasma creatinine; n = 3 (**B**) *ngal* mRNA; n = 5 (**C**) ngal and Kim-1 protein expression; n = 3 (**D**) Densitometry analyses of ngal and Kim-1 normalized to total protein. n = 3 mice/group/day *p < 0.05 vs. d0, ^$^p < 0.05 vs MI-control at respective day. **(E)** Representative PAS stained kidney histology at day 1 post-MI compared with No-MI control, n = 5 **(F)** Representative LV picrosirius red images and collagen density of No-MI, MI-control, Lipo-15-epi-LXA_4_ (1 µg/kg) and -15-epi-LXA_4_ (1 µg/kg) injected mice at d5 post-MI; n = 5 (**G**) Bar graph representing percentage stained area of collagen at d5 post-MI. n = 5 mice/group/day (**H**) LVI α-SMA protein expression; n = 3 (**I**) Densitometric analysis of α-SMA immunoblot *p < 0.05 vs. d0, ^$^p < 0.05 vs MI-control at respective day.

Post-MI, the injured LV drives immediate renal inflammation leading to chronic kidney disease. Thus, we evaluated the effect of 15-epi-LXA_4_ on the cardiorenal axis. Though there was no significant difference in creatinine levels in the no-MI control, post-MI control and 15-epi-LXA_4_ injected mice (Fig. 6A). Post-MI renal inflammation was confirmed by early upregulation of *ngal* (17 fold, p < 0.01), at post-MI d1 and 4.0 fold at post-MI d5 in MI-control. 15-epi-LXA_4_ inhibited ngal expression at d1 post-MI compared to respective MI-control. Further, at d5 post-MI there was an increase in acute kidney injury marker Kim-1 in MI control indicating cardiorenal inflammation which was inhibited in the 15-epi-LXA_4_ group (Fig. 6B–D). The PAS staining of renal cortex displayed distortion of Bowman’s capsule with thickening of glomerular basement membrane in MI-control group at d1 post-MI. Both Lipo- and 15-epi-LXA_4_ injected mice show limited changes in glomerular structure post-MI d1 (Fig. 6E). Post-MI, collagen deposition occurs in response to the acute inflammatory response in the infarcted LV. Quantitative analyses of infarcted area collagen density stained using picrosirius red revealed that there was increased collagen density in response to MI in controls and lipo- and 15-epi-LXA_4_- injected mice (Fig. 6F,G). Of note, 15-epi-LXA_4_ injected mice displayed a decrease in α-SMA expression compared to MI-control group at d5 post-MI (Fig. 6H and I) indicated non-canonical pathway regulation in 15-epi-LXA_4_ treated mice compared with MI-control mice. Thus, post-MI treatment of 15-epi-LXA_4_ limited acute inflammation in the kidney with no effect on LV collagen density post-MI.

## Discussion

Complications in post-MI acute healing lead to recurrent ischemic, mechanical, arrhythmic, and inflammatory disturbances, which together account for a majority of fatalities that occur in heart failure patients^10^. Anti-inflammatory agents, including tumor-necrosis factor inhibitors, impair the resolution of inflammation and promote heart failure^13^. Therefore, novel alternative treatments for delaying heart failure could be attempted to resolve post-MI inflammation by using proresolving mediators like 15-epi-LXA_4_. Endogenous bioactive 15-epi-LXA_4_ is a stable analog of LXA_4_ produced by acetylation of COX-2^16^. The presented results show that 15-epi-LXA_4_ improved LV function by early activation of the resolving phase post-MI. The key evidence of early activation of the resolving phase with unaltered innate response are: 1) increased FPR2 and Ccl2 thereby leading to rapid neutrophil clearance by d5 post-MI; 2) reduced proinflammatory response at LV and remote organs, i.e. spleen and kidney; and 3) activated GPR120 and inhibited GPR40, along with increased proresolving markers during the resolving phase post-MI. Thus, the present study revealed that bioactive 15-epi-LXA_4_ exerts proresolving properties and thus has potential that demands further long-term exploration in the chronic heart failure setting.

Resolution of inflammation is an active process regulated by leukocytes (neutrophils, monocytes/macrophages) and some other proresolving lipid mediators^13, 14^. Fatty acid receptors are known to play a role in the resolution of inflammation and FPR2 is a receptor for 15-epi-LXA_4_. GPR120, GPR40, and FPR2 are highly expressed on macrophages, thus based on expression pattern, GPR40, GPR120, and FPR2 emerged as a receptor of particular interest in achieving the resolution of inflammation post-MI. Lipoxins and their 15-epimers are both known to exert proresolving responses through activation of GPCR^26^. Many reports suggest that FPR2 is present on neutrophils, monocytes, and macrophages in order to regulate chemotaxis^27, 28^. In the current study, we further delineated the macrophage class switching plasticity using 15-epi-LXA_4_ and interaction with GPCR not only on FPR2 but also with GPR40 and GPR120. First, *in-vitro* results indicated classical (M1) macrophages expressed FPR2 and GPR40 while alternative (M2) macrophages expressed FPR2, along with GPR120 and a lower expression of GPR40. GPR40 acted as a functional sensor to promote the acute inflammatory response, while GPR120 promoted the resolution process. Since, 15-epi-LXA_4_ acts as a sensor for FPR2 and GPR120, while also being an inhibitor for GPR40 suggests that 15-epi-LXA_4_ monitors resolution receptors. Second, *in-vitro* results are further validated *in-vivo* post-MI studies where 15-epi-LXA_4_ in both liposomal and free form activated FPR2 at d1 post-MI. Several *in-vitro*, pre-clinical and clinical human studies have shown that LXA_4_ acts as an agonist to FPR2 in order to exert a proresolving response^29–31^. Our study indicates that 15-epi-LXA_4_ also acts as an agonist to GPR120 while being an antagonist for GPR40 to heal the infarcted LV and reduce post-MI LV dysfunction. In response to inflammation, a diverse nature of chemo-attractants regulates immune cell kinetics with sequential modification of acute and healing phases at the inflammatory sites^32^. In this post-MI study, the 15-epi-LXA_4_ treated mice activated Ccl2 simultaneously with FPR2 at d1 post-MI, which enables leukocyte trafficking to the inflammatory site, i.e. LV but also at remote organs such as the spleen and kidneys. Thus, 15-epi-LXA_4_ mediated the simultaneous early activation of FPR2 and Ccl2, suggesting a robust activation of the innate inflammatory phase, which is essential for the physiological healing of the inflammatory response post-MI.

15-epi-LXA_4_ inhibits chemotaxis, adherence, and transmigration of neutrophils along with inhibiting neutrophil–epithelial and endothelial cell interactions^33, 34^. Post-MI, 15-epi-LXA_4_ promoted LV healing through increased neutrophil infiltration in the acute phase and during the resolution phase promotion of the rapid clearance of neutrophils. *In-vitro* and *in-vivo* results provide evidence that 15-epi-LXA_4_ promotes the clearance of neutrophils in order to achieve the early initiation of the resolving phase thereby shorten the inflammatory phase. Lipoxins (lipoxygenase interaction products; e.g. LXA_4_) are endogenous bioactive molecules generated by transcellular metabolism and are actively known to promote resolution pathway like other proresolving lipid mediators, such as resolvins^15^. Like LXA_4_, Resolvin D1 interacts with FPR2 and GPR32 that are responsible to mediate the proresolving signal^35^. Similarly, 15-epi-LXA_4_ mediates the proresolving signal through FPR2, GPR40, and GPR120. 15-epi-LXA_4_ shortens the proinflammatory window without altering the acute inflammatory response that is required immediately after MI for phagocytic clearance of necrotic or apoptotic myocyte^36^. Since the active resolution of inflammation depends on a successful and coordinated transition from the initial recruitment of neutrophils to the more sustained population of mononuclear cells. Post–MI, increased levels of cytokines IL-1β and IL-6 at d1 modulates the inflammation axis. IL-6 represents a checkpoint regulator of neutrophil trafficking while IL-1β regulates neutrophil migration during the inflammatory response to coordinate resolution of inflammation^37, 38^. Post-MI, IL-6 is produced by some different cell types including monocytes, macrophages, and fibroblast while IL-1β is produced by monocytes and macrophages, both contributing to the active inflammatory response and heart failure pathology^39^. Thus, 15-epi-LXA_4_ coordinates the upregulation of IL-6 and IL-1β at d1 to facilitate an unaltered active inflammatory phase post-MI.

Early activation of the resolving phase following the innate response determines successful healing to control an overactive and unresolved inflammation post-MI. The capacity of macrophages to sequentially exhibit predominant anti-inflammatory properties than classical phenotypes defines the resolution process. Macrophages are a multifunctional diverse cell type, to simplify the resolution process in healing, for easiness macrophages are defined as M1 when possessing a proinflammatory role, and M2, when possessing tissue repair and proresolving functions. Early activation of the resolution process in 15-epi-LXA_4_ treated mice was supported by a consistent increase in *Mrc-1*, *Ym-1*, and *Arg-1* post-MI. Though the lipo-15-epi-LXA_4_ injected mice did display an increase in *Ym-1* and *Arg-1* at d1; this could be due to a slow release of 15-epi-LXA_4_ from liposomes.

Renal dysfunction is important in post-MI comorbidities for heart failure with diminished kidney function. The interaction between heart failure and kidney dysfunction is bidirectional to acute or chronic dysfunction of the heart and vice versa. NGAL is a potent acute kidney injury biomarker, which is immediately elevated within 24 hr post-MI as a result of the acute inflammatory and cardiorenal axis^40^. Similarly, Kim-1 is another biomarker that validates renal injury^41^. Increased *ngal* at d1 and Kim-1 at d5 post-MI displays bilateral and MI-mediated collateral renal cortex injury. Post-MI decreased *ngal* expression in 15-epi-LXA_4_ treated mice suggests that 15-epi-LXA_4_ limited excess neutrophil infiltration, not only at the site of injury (LV) but also in remote organs such as the kidneys. Post-MI reduced expression of Kim-1 at d5 in 15-epi-LXA_4_ mice suggested lower renal inflammation. Thus, 15-epi-LXA_4_ could be an immunoresolvent to delay heart failure with post-MI renal protective properties. A clinical trial using 15-epi-LXA_4_ suggest positive results to inhibit the skin inflammatory lesions of infantile eczema, and indicate safety for the use of LXA_4_ analogs in clinical treatment^42^. Post-MI treatment with 15-epi-LXA_4_ decreased α-SMA without affecting collagen density suggested the non-canonical pathway regulation in 15-epi-LXA_4_ treated groups. Though the fibrosis was unchanged, but reduced infarct area expansion in 15-epi-LXA_4_ treated mice, displaying limited LV hypertrophy indicated feed-forward loop regulation in the resolution of inflammation. Post-MI 15-epi-LXA_4_ clears neutrophils rapidly from the infarcted wall, thereby resolving inflammation and reduced infarct area expansion compared to non-treated mice. The presence of neutrophils in the infarcted wall at day 5 post-MI displayed signs of unresolved inflammation post-MI. Thus, the possible mechanism of 15-epi-LXA_4_ is helping to build less inflamed stable extracellular matrix due to early turn-off inflammation with immediate turn-on resolution post-MI^43^. The current study monitored the proresolving capacity of 15-epi-LXA_4_ in response to acute post-MI healing, the long-term studies are warranted to validate the role of 15-epi-LXA_4_ in chronic heart failure.

Our study has highlighted the significant advantages for liposomal drug delivery, achieved from the liposomes’ controllable size ranging 10–200 nm, which enhances penetration into blood vessels and helps to reduce systemic degradation of biomolecules compared to a non-liposomal or free drug^44, 45^. More importantly, liposomal drug delivery for small bioactive molecules, such as 15-epi-LXA_4_ provides the protection in the blood stream during circulation that avoids the fast clearance from the reticulo-endothelial system and depresses the immune response before reaching the targeting tissues^46, 47^. Liposomal delivery of 15-epi-LXA_4_ showed similar protective function as free 15-epi-LXA_4_, improving ventricular function to a similar extent. Furthermore, liposomal delivery of 15-epi-LXA_4_ limited changes in the proinflammatory markers IL-6 and IL-1β in the remote organs spleen and kidneys (Fig. 3D and G) compared to free 15-epi-LXA_4_ delivery.

**Figure 7:**
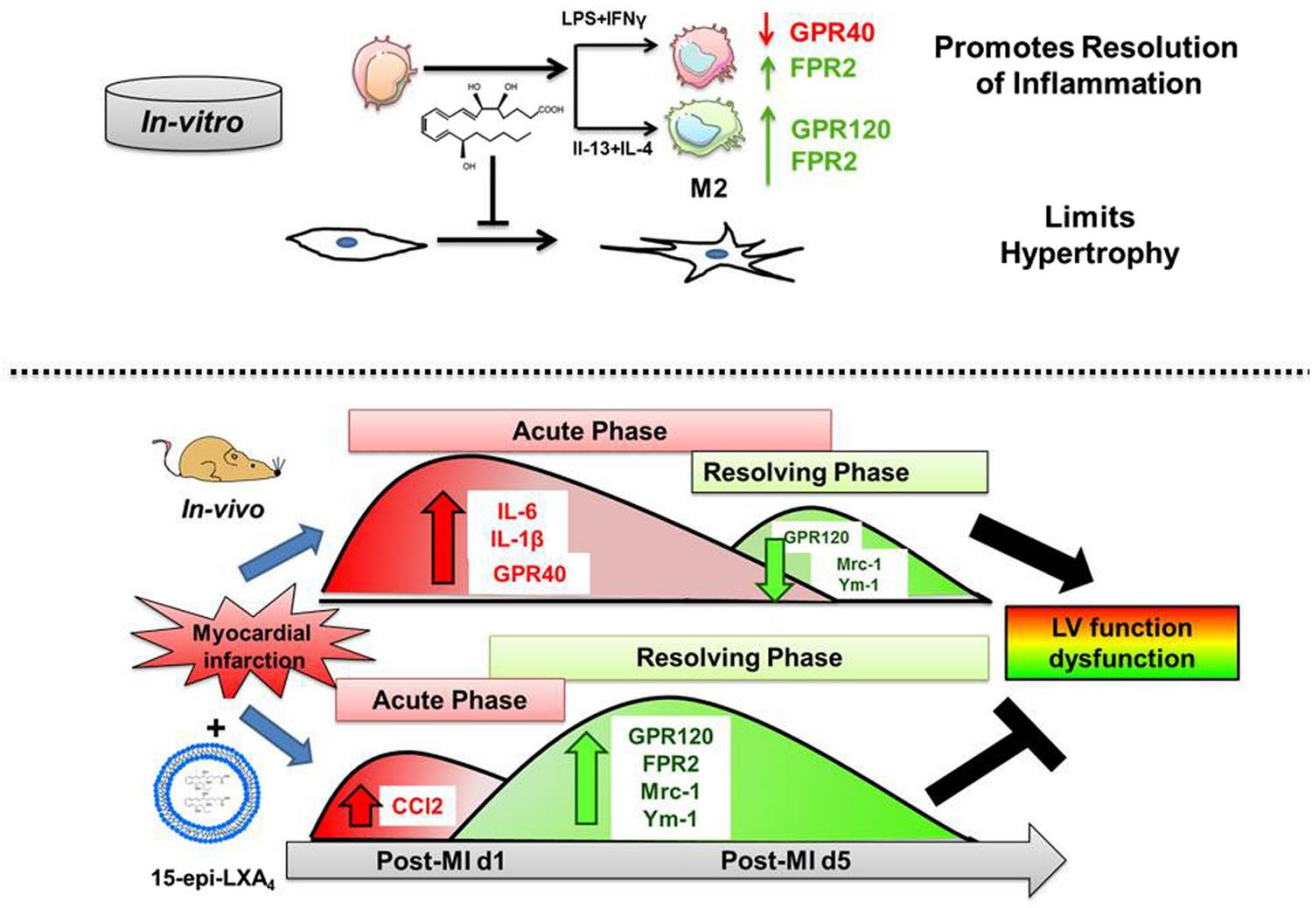
Schematic summary: 15-epi-LXA_4_-mediates early activation of resolving phase. The upper panel displays the mechanism of 15-epi-LXA_4_ on macrophage phenotype and fibroblast differentiation *in-vitro* and the lower panel shows 15-epi-LXA_4_-mediated early activation of resolving phase post-MI. Cell shapes are used from Servier Medical Art (www.servier.com), licensed under a Creative Commons 3.0.

In conclusion, we demonstrated that 15-epi-LXA_4_ initiates early activation of the resolving phase (Fig. 7) thereby improving LV function post-MI. 15-epi-LXA_4_ activated neutrophil clearance in the LV during the resolving phase with an increase in FPR2 and Ccl2 during the acute inflammatory phase. Additionally, 15-epi-LXA_4_ promotes the resolution of inflammation by activating GPR120 and inhibiting GPR40. 15-epi-LXA_4_ potentially acts on the cardiorenal axis by inhibiting kidney *ngal* and Kim-1 expression post-MI. In summary, our findings indicate that 15-epi-LXA_4_ possesses the high therapeutic potential to modulate neutrophil biology and resolution physiology to limit cardiac remodeling, thus regulating renal pathology and thereby delaying cardiorenal failure post-MI.

## Material and Methods

### Materials

Egg phosphatidylcholine (EPC) and (DSPE-PEG (2000)) (1,2- distearoyl-sn-glycero-3-phosphoethanolamine (polyethylene glycol)- 2000) were obtained from Avanti Polar Lipids (Alabaster, AL, USA). Cholesterol was purchased from Sigma (St. Louis, MO, USA) and 5(S),6(R),15(R)-trihydroxy-7E,9E,11Z,13E-eicosatetraenoic acid (15-epi-LipoxinA_4_) was obtained from Cayman Chemicals (Ann Arbor, MI, USA).

### Animal care and compliance

All animal MI surgery procedures and treatments were conducted according to the “Guide for the Care and Use of Laboratory Animals” (8th Edition. 2011), and AVMA Guidelines for the Euthanasia of Animals: (2013 Edition) and were approved by the Institutional Animal Care and Use Committees at the University of Alabama at Birmingham, USA.

### Macrophages isolation and 15-epi-LXA_4_ treatment

Peritoneal macrophages (PM) were isolated from C57BL/6 J mice as previously described^48^ with slight modifications. Briefly, the mice were anesthetized with 2% isoflurane using a tabletop anesthesia machine (SurgiVet^TM^), and the peritoneal cavity was lavaged twice with 10 ml of ice-cold RPMI 1640 media with 10% FBS) and 1% antibiotics. The recovered media was centrifuged at 250 x g for 10 min. The cell pellet was re-suspended in 6 ml of RPMI 1640 media. The cells were plated in a 6-well plate (1 × 10^6^ cells/ well), incubated at 37 °C overnight to allow the cells to adhere, and subsequently washed with fresh media to remove any unattached cells. To differentiate PM into M1 or M2 phenotype, the cells were stimulated with LPS (1 µg/ml) + IFNγ (20 ng/ml) and IL-4 (20 ng/ml) + IL-13 (20 ng/ml) respectively for 4 hrs. Both M1 and M2 macrophages were treated with 15-epi-LXA_4_ (100 nM) for 2 hrs.

### Cardiac fibroblast isolation, TGF-β-induced differentiation, and 15-epi-LXA_4_ treatment

The mice were anesthetized using 2% isoflurane, and the chest cavity was opened to remove the heart. After heart isolation, the atria, and right ventricle were removed, and the LV was minced using surgical blades. Cardiac fibroblasts (CF) from the LV were isolated by enzymatic digestion with 600 U/ml collagenase II and 60 U/ml DNase I. Cells at passage 2 were plated in 6-well plates (5 × 10^4^ cells/well) and allowed to attach at 37 °C overnight, then washed using DMEM/F-12 media with 10% FBS and 1% antibiotics to remove unattached cells. The cells were plated on Millicell EZ slide-8-well (Millipore). The cells were serum deprived for 24 hrs and then stimulated with TGF-β (15ng/ml) for 18hrs for myofibroblast differentiation and co-incubated with 15-epi-LXA_4_ (100 nM). Cells were fixed using 4% PFA (paraformaldehyde), permeated using 0.1% Triton and blocked for 1hr in 10% goat serum. Cells were subsequently incubated with mouse monoclonal anti-α-SMA antibody (Sigma) overnight and Alexa 555-labeled anti-mouse antibody (Molecular Probes), each for 60 min at room temperature. The nucleus was stained using DAPI (Molecular Probes). Cells were mounted using an anti-fade mounting media (Invitrogen) and then visualized and photographed using a Nikon A1 High Speed Laser Confocal microscope. Total 20 cells were counted per field by the morphology i.e., spindle shape vs stellate. Total 5 fields were counted for each group.

### Liposomal preparation and quality control measurement of 15-epi-LXA_4_ loaded liposomes

Liposomes loaded with 15-epi-LXA_4_ were prepared by hydration of thin films (EPC/DSPE-PEG2000/cholesterol, 23:16:1 mol%) with 5 µg/ml 15-epi-LXA_4_ and PBS (1X, HyClone) solutions, followed by sonication and stirring. The prepared liposomes were sized by repeated extrusions (25 °C) through polycarbonate filters with pore diameters of 0.05 µm (Fisher Scientific, USA). The extruded 15-epi-LXA_4_ loaded liposomes were then dialyzed in a 1 ml, ready-to-use dialysis tube (MWCO at 3000 Da, Spectrum Labs) in PBS at 4 °C for 6 hours to release unloaded 15-epi-LXA_4_. To ensure quality control, total 15-epi-LXA_4_ loading amount was quantified based on the weight of LXA_4_ in liposomes and analyzed by UV-visible spectroscopy (Varian Cary 50, USA). Dialyzed 15-epi-LXA_4_ liposomes were freeze-dried and re-dissolved in methanol/PBS (9:1 Vol %) to extract 15-epi-LXA_4_. The extracted solution was analyzed using UV-vis spectroscopy and quantified based on a pre-established calibration curve. For morphometric quality control, liposome size and distribution were analyzed with Dynamic Light Scattering (DLS) using Nano-ZS Zetasizer (Malvern). The average hydrodynamic particle size was determined from three independent runs. Standard deviation (PDI) was obtained to evaluate the size distribution.

### Coronary ligation surgery and 15-epi-LXA_4_ treatment

8–12 weeks C57BL/6 J mice were obtained from The Jackson Laboratory (Bar Harbor, Maine, USA) and were maintained under constant temperature (19.8–22.2 °C). In brief, the mice were anesthetized with 2% isoflurane, and the left anterior descending coronary artery was permanently ligated using prolene 6–0. At 3 hours post MI surgery, mice were injected with either lipo-15-epi-LXA_4_ (1 µg/kg/day; SQ) or free15-epi-LXA_4_ (1 µg/kg/day; SQ) till MI-day one (d-1) or till d5 post-MI. The mice were given free access to water and standard chow diet. The mice were divided into 4 groups: (1) No surgery (day 0 naïve control: No-MI control) (2) MI-control group. (3) Liposomal-15-epi-LXA_4_ (Lipo-15-epi-LXA_4_) (4) 15-epi-LXA_4_ (15-epi-LXA_4_).

### LV function measurements by echocardiography

LV function was measured using the Vevo 770 imaging system (VisualSonics Inc.), as described previously^49^.

### Necropsy and infarct area analysis

No-MI control day (d0), d1 or d5 post-MI 15-epi-LXA_4_ injected and untreated mice were anesthetized with isoflurane briefly, then mice were under 2% isoflurane anesthesia in 100% oxygen mix, and heparin (4 IU/g) was injected. The blood was collected from the carotid artery and centrifuged for 5 min to separate plasma. The spleen, lungs, LV and right ventricle were separated and weighed individually. The LV was divided into apex (infarcted area), mid-cavity, and base (remote area) under a microscope. The spleen and kidney are collected, weighed and fixed in 10% zinc formalin for immunohistochemistry (IHC) and molecular analysis as described previously^15^.

### Quantitative real-time PCR for measurements of gene transcripts

For qPCR, reverse transcription was performed with 2.0 μg of total RNA using SuperScript® VILO cDNA Synthesis Kit (Invitrogen, CA, USA). Quantitative PCR for *FPR2*, *IL-6, ccl2, IL-1β, Arg-1, Mrc-1, Ym-1, neutrophil gelatinase-associated lipocalin (ngal)*, *GPR40* and *GPR120* genes was performed using taqman probes (Applied Biosystems, CA, USA) on master cycler ABI, 7900HT. Gene levels were normalized to *Hprt-1* as the housekeeping control gene. The results were reported as 2^−ΔCt^ (ΔΔCt) values. All the experiments were performed in duplicates with n = 5/group.

### LV histology and immunohistochemistry and confocal microscopy

For histological measurements, LV transverse section was embedded in paraffin and sectioned. The assessment of neutrophils and macrophages by immunohistochemistry was done as previously described^49^. The confocal microscopy was performed om LV transverse frozan section using F4/80 and MRC-1 (abcam) as previously described^50^.

### Histological Analysis of Kidneys using PAS staining

The kidneys from no-MI, MI-control, Lipo-15-epi-LXA_4_ and 15-epi-LXA_4_ injected mice were collected and weighed. Longitudinal middle slice of the kidney, taken through the hilum, was embedded in paraffin and stained with periodic acid-Schiff (PAS) reagent.

### Picrosirius red staining

Post-MI collagen measurements were done by picro sirius red (PSR) staining as described previously^15^.

### Image analysis for Immunohistochemistry and PSR staining

For each slide per mouse, a total of 5–7 images were acquired from LV infarcted area including border zone as previously described^15^.

### Plasma Creatinine measurement

Plasma creatinine levels from No-MI (d0), MI-control and 15-epi-LXA_4_, injected mice at post-MI d1, and d5 were determined using LC-MS/MS. Briefly, 10 µl of sample was deproteinated and diluted with heavy isotope-labeled internal standard (ISTD) in a single step by adding ISTD in 80% acetonitrile. Twenty microliters of diluted sample is subjected to isocratic, HILIC HPLC with 10 mM ammonium acetate in 65% acetonitrile at the rate of 0.15 ml/min. Creatinine and d3-creatinine (ISTD) are detected by electrospray ionization tandem mass spectrometry (Quattro Micro API) MRM transitions 114 > 44 and 117 > 47, respectively. Quantitation is achieved by comparing results to a synthetic standard calibration curve (0, 0.2, 1, 5, 100 µg/ml for serum).

### LV protein extraction for immunoblotting

The left ventricle infarct (LVI) tissues were processed for protein extraction as previously described^15^.

### LV protein Immunoblotting

Electrophoresis of 10 µg of LVI protein as previously described^15^ and probed with primary antibody (FPR2; 1:1000 and GPR40; 1:1000) overnight at 4 °C followed by secondary antibody (Biorad). The proteins were detected using femto chemiluminescence detection system (Pierce Chemical, Rockford, IL, USA). Densitometry was performed using Image J software (NIH, USA).

### Statistical analysis

Data are expressed as mean and SEM. Statistical analyses were performed using Graphpad Prism 5. Analysis of variance (One way ANOVA) followed by Newman–Keuls post-hoc test was used for multiple comparisons between post-MI d1, d5 MI-control and 15-epi-LXA_4_ injected groups and No-MI control. All immunoblotting densitometry data were normalized to total protein/lane. p < 0.05 was considered as statistically significant.

## Electronic supplementary material

Suppl info
