# Supplementary material for: Resolution Agonist 15-epi-Lipoxin A4 Programs Early Activation of Resolving Phase in Post-Myocardial Infarction Healing
Source: Sci Rep. 2017 Aug 30;7:9999. doi: 10.1038/s41598-017-10441-8 (PMC5577033; doi:10.1038/s41598-017-10441-8)

Resolution Agonist 15-epi-Lipoxin A_4 Programs Early Activation of Resolving Phase in Post-Myocardial Infarction Healing

Vasundhara Kain[1], Fei Liu[2], Veronika Kozlovskaya[2], Kevin. A. Ingle[1,], Subhashini Bolisetty[3], Anupam Agarwal[3], Santosh Khedkar[4], Sumanth D. Prabhu[1], Eugenia Kharlampieva[2] and Ganesh V. Halade[1]

[1]Division of Cardiovascular Disease, [2]Department of Chemistry, [3]Division of Nephrology, The University of Alabama at Birmingham, Alabama; [4]ChemBio Discovery Solutions, Lexington Massachusetts.

Supplementary figure legends

Supplementary figure 1. **15-epi-LXA_4 limits TGF-β-mediated myofibroblast differentiation.** Immunofluorescence quantification data selected from total 20 cells that were counted per field on the basis of the morphology. Total 5 fields were counted for each group, the data is represented as stellate: spindle ratio. n=5; *p<0.01 vs. control, $p<0.05 vs 15-epi-$LXA_4$ treatment.

Supplementary figure 2. **15-epi-LXA_4 mediates resolution of inflammation via activation of FPR2 and Ccl2. (A-D)** mRNA expression of *FPR2* and *Ccl2* in spleen and kidney of no-MI, MI-control, Lipo-15-epi-LXA_4 (1 µg/kg) and 15-epi-LXA_4 (1 µg/kg) injected mice at post-MI d1 and d5. n=5 mice/group/day;*p<0.001 vs. d0, $p<0.05 vs MI-control at respective day.

Supplementary figure 3. **15-epi-LXA_4 activated resolving macrophages (A)** Bar graph representing percentage stained area of macrophages at d0 and d5 post-MI. **(B)** Microphotograph of high power field (HPF) macrophage images (60x; yellow) indicating higher expression of Mrc-1 (red) on macrophages (F4/80-green) in 15-epi-LXA_4-injected mice.

Supplementary figure 4. **15-epi-LXA_4 activated resolving macrophages.** Representative immunofluorescence of LVI images showing F4/80 positive macropages (green) that expresses higher MRC-1 (red) in 15-epi-LXA_4-injected mice compared to non-treated mice, (maginfication; 60x) at d5 post-MI. Nuclei are shown in blue by hoescht staining. Images are reprenstative of n=2 mice/group/day.

Supplementary figure 1

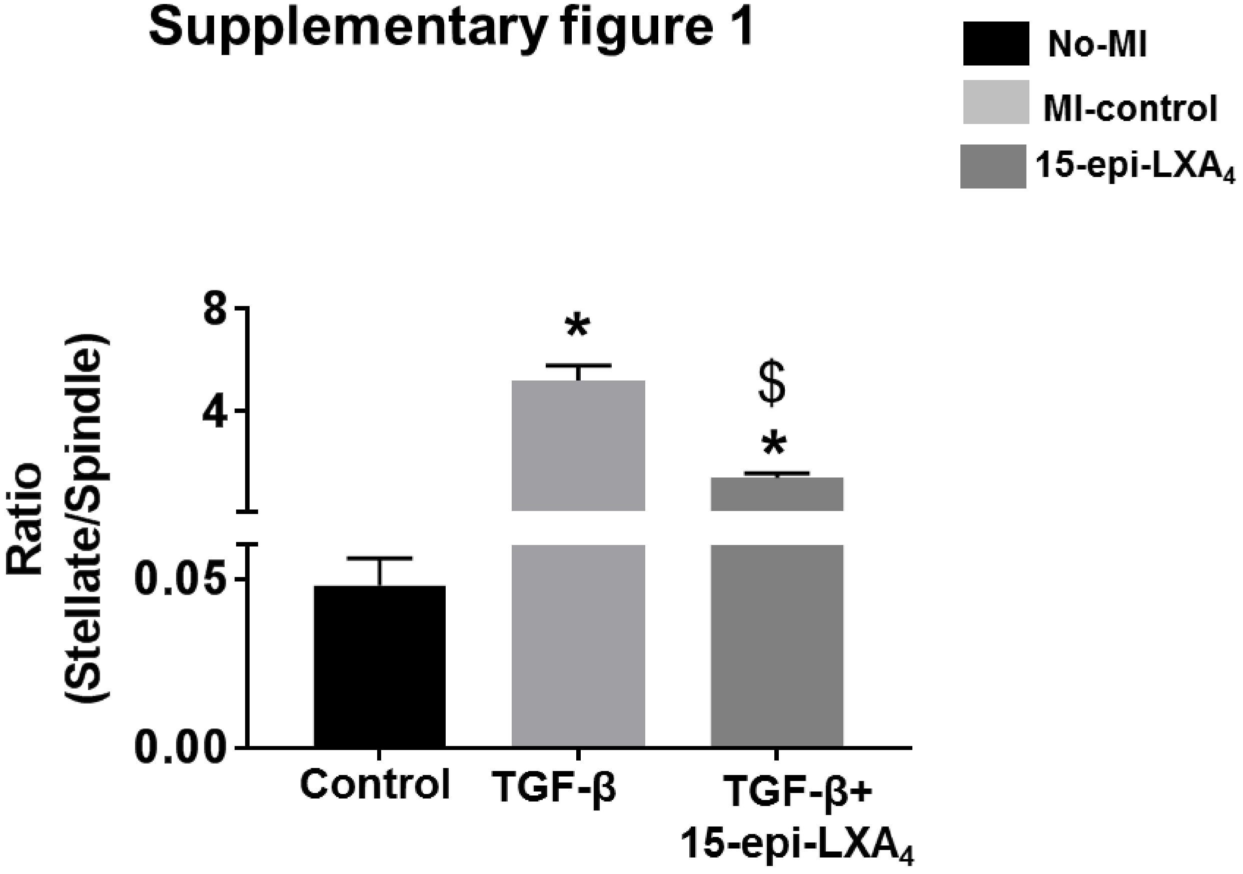

Supplementary figure 2

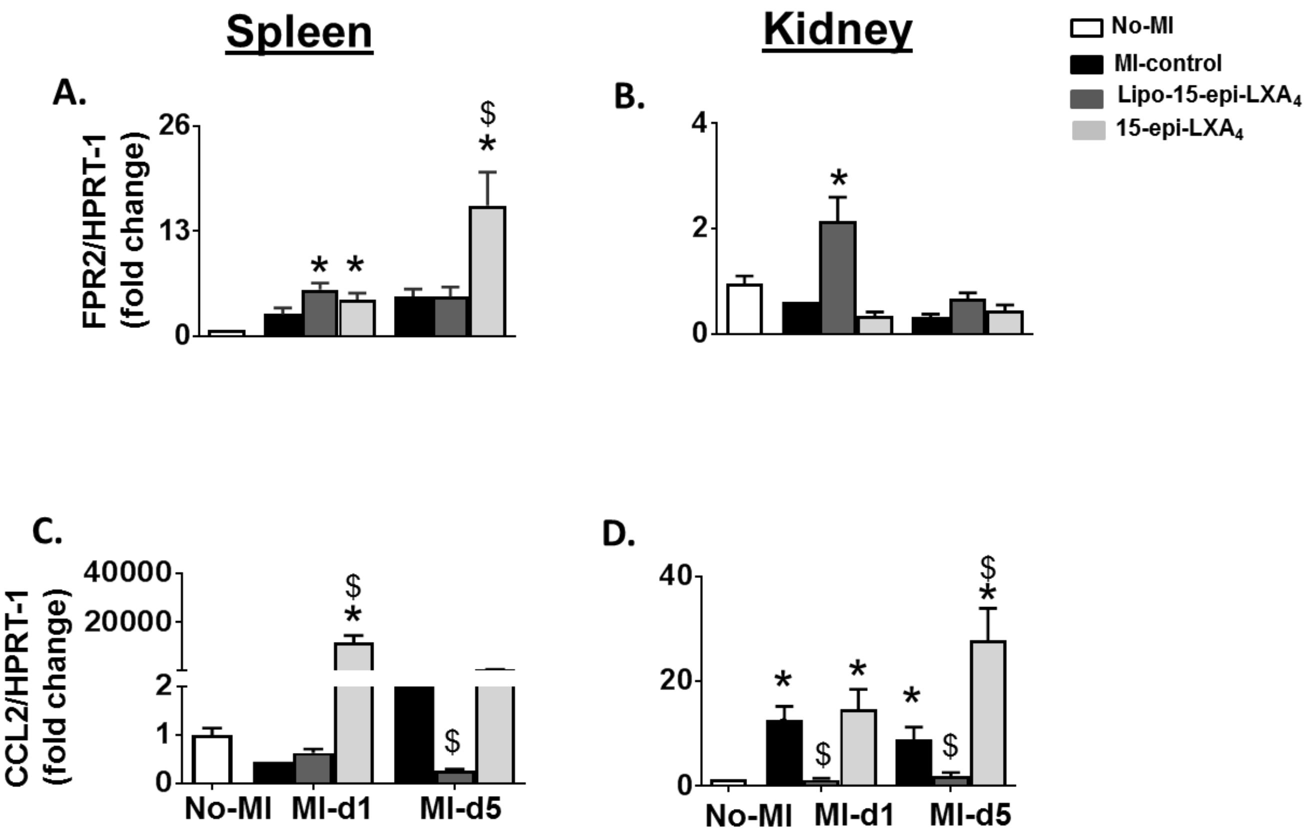

Supplementary figure 3

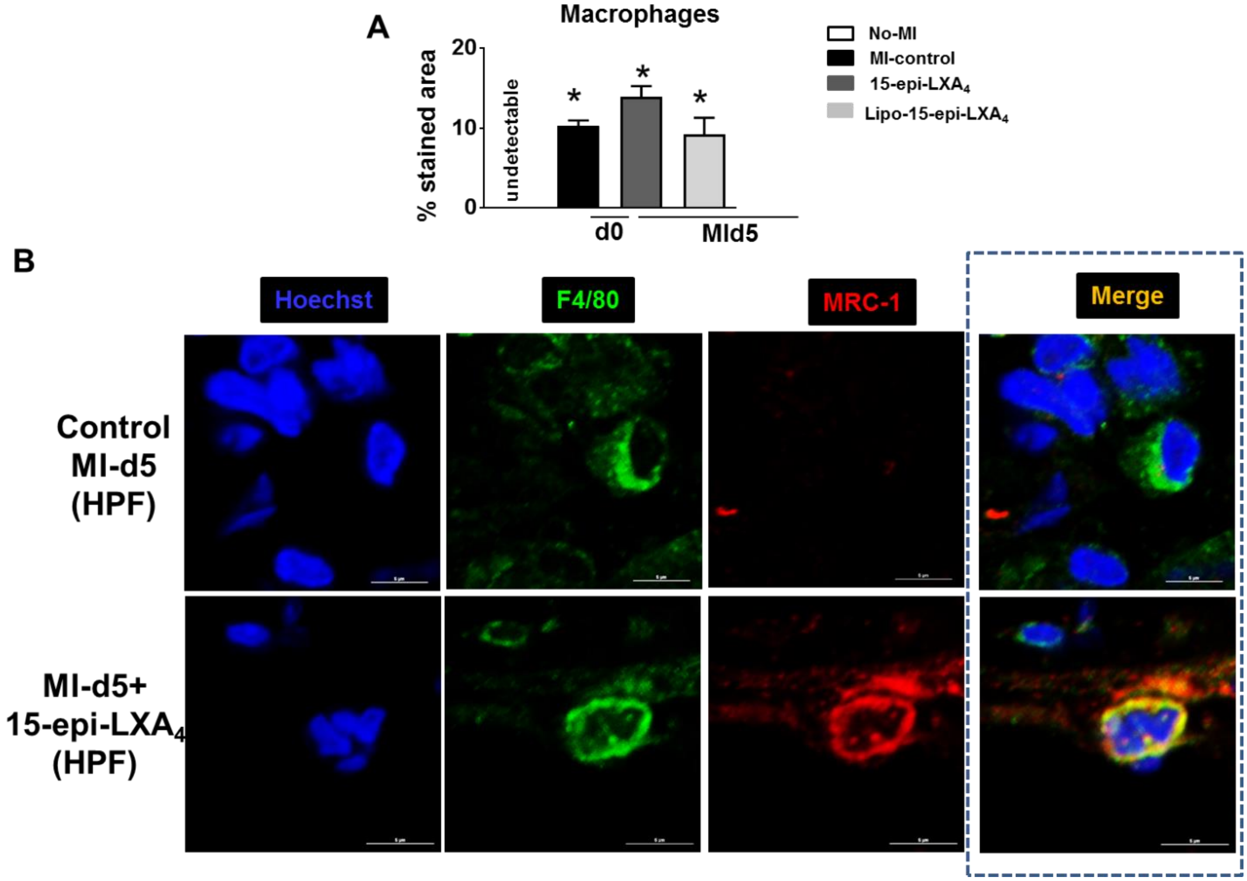

Supplementary figure 4

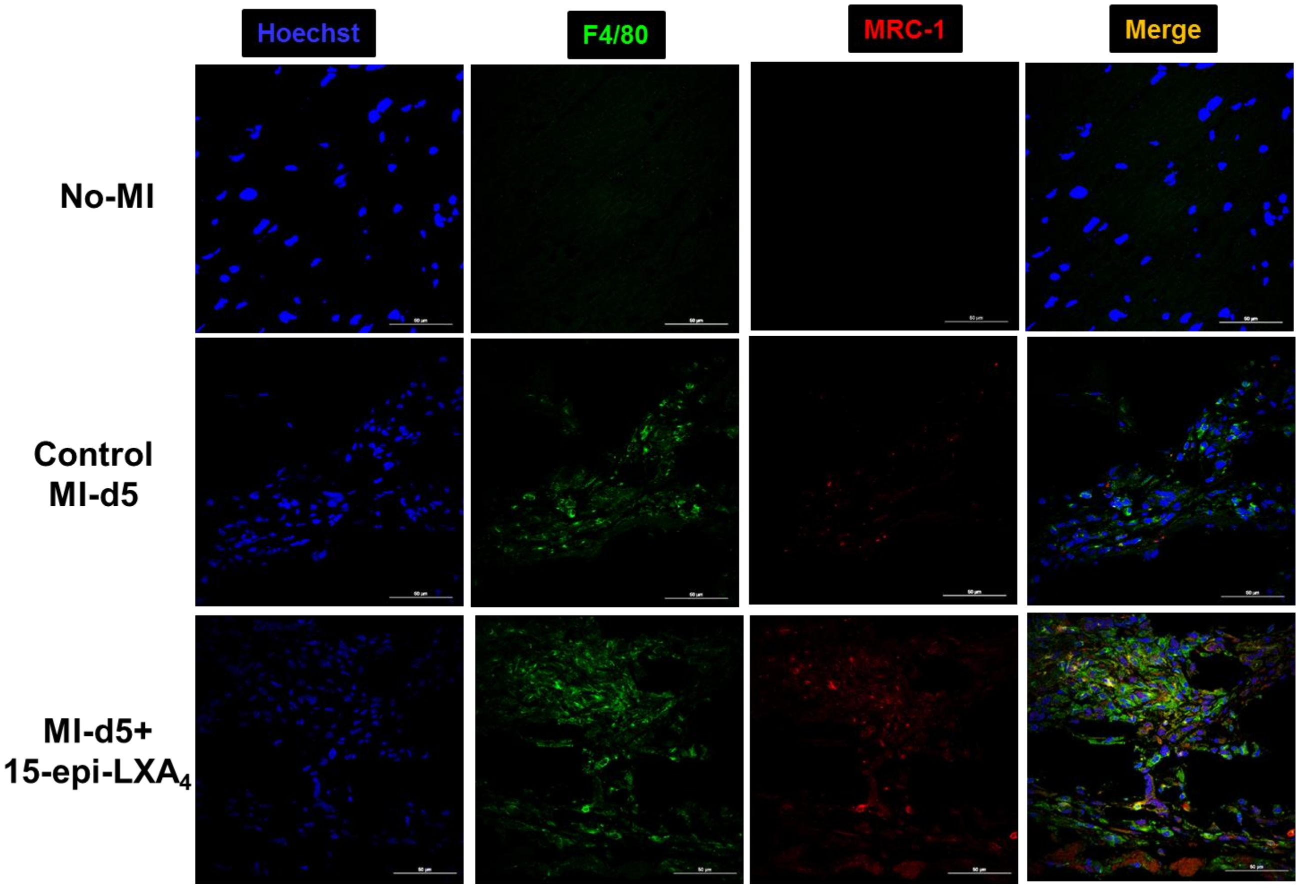

Supplement: Supplementary file 1 — Suppl info [file 41598_2017_10441_MOESM1_ESM.pdf]
